# Supplementary material for: Data Collection for the Fourth Multicentre Confidential Enquiry into Perioperative Equine Fatalities (CEPEF4) Study: New Technology and Preliminary Results
Source: Animals (Basel). 2021 Aug 30;11(9):2549. doi: 10.3390/ani11092549 (PMC8472153; doi:10.3390/ani11092549)
Supplement: Supplementary file 1 [file animals-11-02549-s001.zip › Supplementary Material S2 03.07.2021.pdf]

**Supplementary Material S2**

**R scripts used in the data cleaning phase**

1) To detect the following *empty* variables: EMPTY=which(is.na(DATOS[i]))
    a. Date.
    b. Species: horse, donkey, mule.
    c. Sex: male, female.
    d. Weight: kg.
    e. BCS: normal, fat, thin.
    f. ASA: 1, 2, 3, 4, 5.
    g. Scheduled: scheduled, non scheduled, urgent.
    h. Type: standing, INH, TIVA, PIVA.
    i. Timetable: normal, out of hours.
    j. Reason: description of the procedure.
    k. Anaesthesia duration.
    l. Induction method.
    m. Recovery method.
    n. Surgery duration (except diagnostic procedures).
    o. Recovery duration (except standing sedations and when the patients die or are euthanised during premedication, induction, maintenance or recovery in the recovery box).
    p. Recovery scores (except standing sedations and when the patients die or are euthanised during premedication, induction, maintenance or recovery in the recovery box).

2) To detect the following *inconsistencies* as wrong codified cases:
    a. Type of anaesthesia
       -When coded as INH but with a CRI in the protocol
       DATOS$CRI=="YES" & DATOS$TYPE=="INH" & DATOS$TRIPLE.DRIP==“NO"
       -When coded as PIVA and there is not a CRI in the protocol
       DATOS$CRI=="NO" & DATOS$TYPE==“PIVA"
       -When coded as TIVA but there are not parenteral agents for maintenance

       DATOS$TIVA=="NO" & DATOS$TYPE==“TIVA”
       -When coded as standing sedations but general anaesthetics were used
       DATOS$GA=="YES" & DATOS$TYPE==“STANDING”
    b. Protocol
       -When coded as TIVA but there is an inhalant agent during maintenance
       DATOS$INH_MAIN=="YES" & DATOS$TYPE=="TIVA"
       -When premedication was not coded
       DATOS$PREM_DRUGS=="NONE"
       -When induction in a general anaesthetic was not coded
       DATOS$TYPE != "STANDING" & DATOS$IND_DRUGS=="NONE"
       -When an inhalant agent was not coded in INH or PIVA
       DATOS$TYPE != "STANDING" & DATOS$TYPE != "TIVA" & DATOS$INH_MAIN=="NO"

-When cases were coded as induction with inhalant agents
DATOS$ISOIND =="YES" | DATOS$SEVOIND=="YES" | DATOS$DESFIND=="YES"

c. Monitoring

-When coded as arterial/invasive monitoring but arterial line was not ticked
DATOS$ARTERIAL !="SUCCESS" & DATOS$ARTERIAL.PRESSURE==“YES”

-When invasive blood pressure was ticked in standing sedations
DATOS$ARTERIAL.PRESSURE=="YES" & DATOS$GA =="NO"

-When volatile agents were measured in TIVA
DATOS$INHALATORY.GASES=="YES" & DATOS$TIVA =="YES"

d. Standing surgeries

-When the box $ETCO_2$ was ticked in standing sedations
DATOS$ETCO2=="YES" & DATOS$GA =="NO"

-When the box $FiO_2$ was ticked in standing sedations
DATOS$FIO2=="YES" & DATOS$GA =="NO"

-When the box Vol. ag. was ticked in standing sedations
DATOS$INHALATORY.GASES=="YES" & DATOS$GA =="NO"

Abbreviations: ASA, American Society of Anaesthesiologists; BCS, body condition score; CRI, constant rate infusion; $ETCO_2$, end-tidal carbon dioxide; $FiO_2$, fraction of inspired oxygen; GA, general anaesthesia; INH, inhalation anaesthesia only; PIVA, partial intravenous anaesthesia; PREM, premedication; TIVA, total intravenous anaesthesia.
